# Ni–Fe phosphide deposited carbon felt as free-standing bifunctional catalyst electrode for urea electrolysis

**DOI:** 10.1038/s41598-021-01383-3

**Published:** 2021-11-09

**Authors:** Woo Hyun Yun, Gautam Das, Bohyeon Kim, Bang Ju Park, Hyon Hee Yoon, Young Soo Yoon

**Affiliations:** 1 Department of Chemical and Biological Engineering, Gachon University, Seongnam, Gyeonggi-do 461-701 Republic of Korea; 2 Department of Polymer Science and Engineering, Kyungpook National University, Sangyeok-dong, Buk-gu, Daegu, Korea; 3 Department of Electronic Engineering, Gachon University, Seongnam, Gyeonggi-do 461-701 Republic of Korea; 4 Department of Materials Science and Engineering, Gachon University, Seongnam, Gyeonggi-do 461-701 Republic of Korea

**Keywords:** Chemistry, Energy science and technology, Materials science

## Abstract

A free-standing catalyst electrode for the urea oxidation reaction (UOR) and hydrogen evolution reaction (HER) in a urea electrolysis cell was synthesized by electroplating a Ni–Fe alloy onto carbon felt, followed by phosphidation (P-NiFe@CF). The prepared P-NiFe@CF catalyst consisted of Ni_5_P_4_, NiP_2_, and FeP with 3D flower-like P-NiFe architecture on CF. P-NiFe@CF exhibited excellent electrocatalytic activity for the UOR (demanding only 1.39 V (*vs.* RHE) to achieve 200 mA cm^−2^), and for the HER with a low overpotential of 0.023 V (*vs.* RHE) at 10 mA cm^−2^, indicating its feasibility as a bifunctional catalyst electrode for urea electrolysis. A urea electrolysis cell with P-NiFe@CF as both the free-standing anode and cathode generated a current density of 10 mA cm^−2^ at a cell potential of 1.37 V (*vs.* RHE), which is considerably lower than that of water electrolysis, and also lower than previously reported values. The results indicate that the P-NiFe@CF catalyst electrodes can be used as free-standing bifunctional electrodes for urea electrolyzers.

## Introduction

Hydrogen (H_2_) has recently gained significant attention as a clean energy source because of its high calorific value and environmental friendliness relative to other hydrocarbon-based fuels. Unlike methane reforming, hydrogen production from water splitting is carbon–neutral and is an inexpensive process that generates high purity H_2_^1^. However, the sluggish oxygen evolution reaction (OER) impedes the overall efficacy of the water-splitting reaction. Thus, to overcome the high overpotential of the OER, readily oxidizable species, such as hydrazine, ethanol, and urea have been explored for reducing the overpotential^2^. In particularly, hydrogen production from urea is of interest because urea is non-flammable, non-toxic, and low-cost, and the denitrification of urea-rich wastewater can be achieved with the simultaneous production of H_2_^3–5^. The theoretical potential for water electrolysis is 1.23 V, whereas that for urea electrolysis is as low as 0.37 V; however, the actual electrolysis cell voltages for water and urea are still high^6^. The high overpotential of urea electrolysis is due to the fact that the anodic urea oxidation reaction (UOR) is a complex process that involves 6e^−^ transfers resulting in sluggish kinetics^7–9^. The sluggish kinetics of the UOR remains a challenge for the practical implementation of urea electrolysis. Against this backdrop, a high-performance bifunctional catalytic system for the anodic UOR and cathodic hydrogen evolution reaction (HER) to facilitate urea electrolysis for H_2_ production has been demanded.

Although, noble metal catalysts such as Pt/C, RuO_2,_ and IrO_2_ are usually preferred for urea electrolysis, their high cost and scarcity limit their large-scale use. Recently, Ni-based materials have been studied as bifunctional UOR and HER catalysts as alternatives to noble group metals^5,9–12^. The Ni-catalyzed UOR suffers from the disadvantages of a large overpotential and susceptibility of the catalysts to CO poisoning. Yan et al.^7^ reported that the overpotential of urea electrolysis could be reduced by the incorporating Co or Zn into the Ni structure. Singh et al.^13^ showed that Sn in NiSn facilitated OH^−^ adsorption on NiSn, resulting in stable UOR peak current densities. Yu et al.^14^ reported that Ni–Mo–O nanorod-derived composited catalysts afforded a reduced overpotential and a smaller Tafel slope (i.e. 19 mV) for the UOR, with long-term stability. Several other Ni-based catalysts have been reported, such as Ni(OH)_2_^15^, Ni_2_P/Fe_2_P^1^, Ni nanosheets^16^, Ni_3_S_2_ on Ni foam^17^, NiFeCo^18^, and LaNiO_3_^19^, with good catalytic performance. However, designing bifunctional catalysts that exhibit excellent activity for both the UOR and HER still remains a challenge.

Ni–Fe alloys have shown great potential for water splitting under alkaline conditions^20,21^. It has been reported that Fe in Ni promotes the formation of more conducting NiOOH, which can dramatically enhance the reaction rates^22–25^. In addition, Fe–Ni based oxides^26^, metal–organic frameworks^27^, phosphides^28^, nitrides^29,30^, and chalcogenides^32^ have been evaluated for the OER and HER. These catalysts demonstrated excellent OER activities; however, their overall efficiency still remains low owing to the poor HER performance (requiring over 1.65 V *vs.* RHE to drive a current density of 10 mA cm^−2^). Among the various metal catalysts, phosphides have emerged as competent candidates for the HER in alkaline electrolytes^33^. The negatively charged phosphorous atom in metal phosphides can effectively trap protons during the electrochemical HER process^34^. Furthermore, metal phosphides have good stability over a wide pH range. Recent studies have shown that bimetallic phosphides are attractive choices compared to monometallic phosphides because the incorporation of a second metal modulates the electronic structure^35^. For example, Husam et al. reported NiCoP as a superior bifunctional catalyst for the HER and OER in alkaline media^36^. Several bimetallic phosphides, such as MnNiP, AgCoP, FeNiP, and NiCoP have been reported as efficient bifunctional catalysts^37–46^. While a few studies on monometallic Ni phosphides have been reported^47,48^, there are limited studies on bimetallic phosphides for simultaneous UOR and HER. This may be due to the difficulties that arise from the complicated and uneconomical process of integrating the merits of UOR and HER activity in single bifunctional catalysts for both reactions in the same electrolyte. Commonly, the catalytic process occurs on metal surfaces. In case of powdered materials, the exposed active surface area is relatively low, and the electron/ion transport is limited. Therefore, suitable conducting support materials should be selected for the rational design of efficient electrodes. Most support materials have three-dimensional (3D) structures as support material can offer highly exposed active sites, better permeation of electrolytes, and good electron transport. Recently, the direct growth of metal catalysts on support materials has been widely explored^49–53^. These types of electrodes maintain a high surface area and porous network structure, which are conducive for efficient electron transport, thus enhancing the conductivity.

Thus, in this study, we report a highly scalable and convenient approach for fabricating self-supporting carbon electrodes (e.g., carbon felt) with incorporated P-NiFe as bifunctional electrocatalysts for the UOR and HER. The support is decorated with the metal catalysts by electroplating, followed by phosphorization in phosphorus vapor. This study demonstrates that Ni–Fe bimetal phosphide-decorated carbon felt can be directly used as a bifunctional catalyst electrode with excellent catalytic activity and good stability in urea electrolysis cells.

## Methods

### Materials and synthesis of catalyst electrodes

As obtained carbon felt (CF; Carbon Fiber Co., China) was heated at 1000 °C for 2 h at a rate of 2.5 °C/min under continuous nitrogen purging to remove organic impurities. Afterward, the CF was treated with an acid solution (H_2_SO_4_:HNO_3_:H_2_O in a ratio of 1:1:1) for 24 h at 60 °C. The acid-treated CF was then washed with ethanol and acetone and dried in a vacuum oven overnight at 45 °C. Following the acid treatment, the CF (2.0 × 4.0 cm) was attached to a platinum wire and used as the working electrode, whereas Ni foam was used as the counter electrode. The CF electrode and counter electrode were immersed in the electroplating solutions (Table 1), where the distance between the electrodes was maintained at 1 cm. The electroplating solution was purged with nitrogen for 20 min, after which electrodeposition was carried out using a DC power supply by applying a constant current of 70 mA cm^−2^ for 1 h at 60 °C. The obtained Ni- or Ni–Fe-deposited CF (denoted as Ni@CF or NiFe@CF) was washed with water, and then dried in a vacuum oven.

**Table 1:** Composition of different baths used for deposition.

Samples	Chemicals	Composition (g L^−1^)
Ni@CF	NiSO_4_·7H_2_O	200
	NiCl_2_·6H_2_O	40
	H_3_BO_3_	60
	Na_3_C_6_H_5_O_7_	79
NiFe@CF	NiSO_4_·7H_2_O	200
	NiCl_2_·6H_2_O	40
	H_3_BO_3_	60
	Na_3_C_6_H_5_O_7_	79
	FeCl_2_·6H_2_O	40

The phosphidation of NiFe@CF was then carried out under phosphorus vapor; in which, 1 g of red phosphorus was placed upstream of a porcelain boat, and NiFe@CF (2 × 2 cm) was placed 1 cm downstream. Subsequently, phosphidation was carried out in a tube furnace at 550 °C for 2 h at a rate of 2 °C/min under continuous nitrogen flow. The phosphorized sample is denoted as P-NiFe@CF. A whitish-gray material was obtained after phosphidation.

### Characterization

The morphological characteristics and structures of the samples were studied using a scanning electron microscope (SEM, JEOL JSM-6700F) equipped with an energy-dispersive X-ray spectroscopy (EDX) and a high-resolution transmission electron microscope (HRTEM, JEOL JEM-4010). The XRD measurements were carried out with a Rigaku X-ray diffractometer with Cu Kα radiation (λ = 1.5418 Å) at a scan rate of 2º/min at an operating voltage of 40 keV and 20 mA. X-ray photoelectron spectroscopy (XPS, K-alpha, Thermo VG, U.K.) employing a monochromated Al X-ray source (Al Kα line: 1486.6 eV) was used to obtain the binding energy plots of the samples.

### Electrochemical measurements

The electrochemical activities of the catalyst samples were evaluated by linear sweep voltammetry (LSV), chronoamperometry (CA), and electrochemical impedance spectroscopy (EIS) using a potentiostat–galvanostat (SP-240, Bio-Logic, France). The EIS profiles of the samples were acquired in the frequency range of 100 kHz to 100 µHz. A conventional three-electrode system was used for the electrochemical measurements. The as-prepared freestanding electrode was directly used as the working electrode; and Hg/HgO (1 M NaOH) and a platinum wire were used as reference and counter electrodes, respectively. All LSV plots were obtained in aqueous KOH under ambient conditions at a scan rate of 5 mV s^−1^. CA measurements of Ni@CF, Ni–Fe@CF, and P-NiFe@CF were performed in 0.33 M urea in 1 M KOH under an applied voltage of 1.43 and − 0.276 V (*vs.* RHE) to evaluate the UOR and HER performances, respectively. The data set was calibrated with respect to the reversible hydrogen electrode (RHE) (*E*_*RHE*_ = *E*_*Hg/HgO*_ + 0.098 + 0.059 × pH).

## Results and discussion

### Characterization

**Figure 1:**
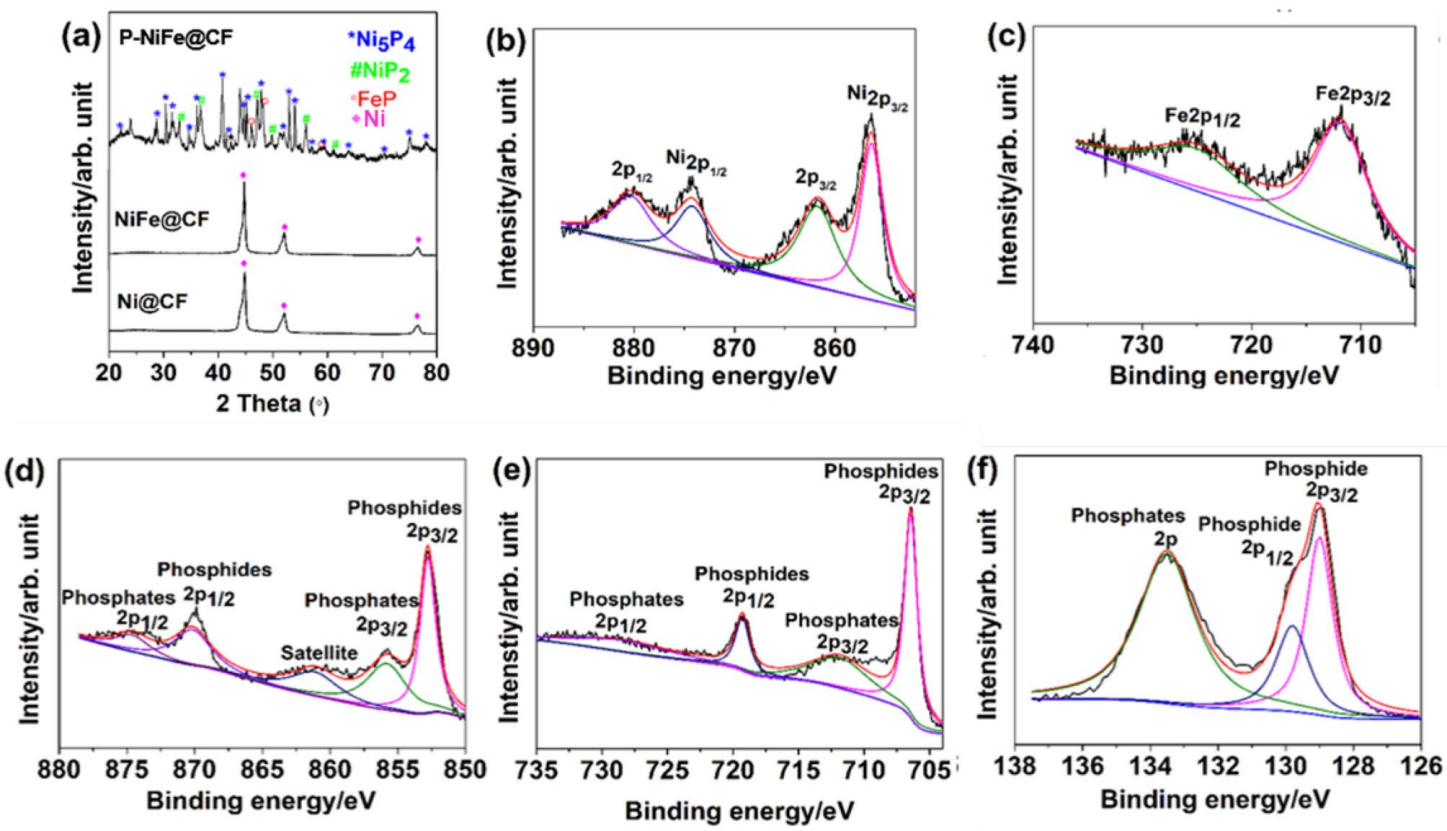
(**a**) XRD patterns of Ni@CF, Ni–Fe@CF, and P-NiFe@CF; and XPS binding energy plots for (**b**) Ni2p and (**c**) Fe2p peaks of Ni–Fe@CF, and Ni2p (**d**), Fe2p (**e**), and P2p (**f**) peaks of P-NiFe@CF.

The XRD patterns of Ni@CF, NiFe@CF, and P-NiFe@CF are shown in Fig. 1a. Ni@CF and Ni–Fe@CF showed well-defined XRD patterns which can be indexed to the fcc structure of Ni (PDF# 98-005-3809)^5,14^. However, no peaks corresponding to Fe were observed in the XRD pattern of Ni–Fe@CF, indicating the substitution of Fe with Ni having the fcc structure. The existence of Fe in NiFe@CF was confirmed by XPS as shown in Fig. 1b. The XRD pattern of P-NiFe@CF exhibited peaks both for both cubic NiP_2_ (PDF#98-002-2221) and hexagonal Ni_5_P_4_ (PDF#98-010-8462). Additionally, the peaks at 46.20°, 48.23°, and 58.96°, ascribed to orthorhombic FeP, were also observed in the XRD pattern of P-NiFe@CF^49^. These results indicated that the P-NiFe@CF catalyst was successfully phosphodized and consisted of NiP_2_, Ni_5_P_4_, and FeP.

The surface elemental composition and valence state of the NiFe@CF and P-NiFe@CF catalysts were analyzed by XPS. Detailed scans of the Ni2p, Fe2p, and P2p regions are shown in Fig. 1b–f. For Ni–Fe@CF, the Ni2p high-resolution spectra displayed peaks at binding energies of 856.4 and 874.3 eV, which were assigned to the Ni2p_3/2_ and Ni2p_1/2_ states, respectively, indicating the existence of Ni^2+^ and Ni^3+^ ions and thus partial oxidation of Ni at the surface^50^. The satellite peaks corresponding to Ni 2p_3/2_ and 2p_1/2_ spin–orbit couplings were observed at 861.7 and 880.15 eV, respectively^51^. Additionally, the Fe2p peaks of NiFe@CF (Fig. 1c) located at 712.1 and 725.4 eV were ascribed to Fe 2p_3/2_ and 2p_1/2_, respectively, indicating the successful incorporation of Fe species into the Ni structure, considering the nonexistence of a crystalline structure including Fe (Fig. 1a). In the case of P-NiFe@CF, peaks were observed at 852.78 and 870.16 eV in the Ni2p (Fig. 1d) region, where these values are close to the binding energy of Ni^δ+^ in Ni_5_P_4_ and NiP_2_, respectively; the result, therefore, indicated increased metallicity of P-NiFe@CF as compared to NiFe@CF. Additionally, the peaks at 855.82 and 874.62 eV are due to Ni-PO_x_^38,42^, while the other peak at 861.13 eV is a satellite peak^42^. The deconvoluted Fe2p spectrum (Fig. 1e) revealed Fe2p_3/2_ peaks at 706.43 and 711.80 eV, derived from Fe–P and Fe–O (corresponding to Fe–P–O_x_)^40,42^. Moreover, the peaks at 719.28 and 728.40 eV were assigned to the Fe2p_1/2_ state of Fe–P and Fe–O, respectively^42^. The P2p spectrum displayed two peaks at binding energies of 128.9 and 129.5 eV (Fig. 1f), corresponding to the 2p_3/2_ and 2p_1/2_ states, respectively, suggesting the existence of a strong bond between P^δ−^ and the metal^34,36^. The peak at 133.4 eV corresponds to the PO_4_^3−^ or P_2_O_5_ species originating from the oxidation of phosphorus upon exposure to air^40^. Elemental analysis based on XPS results shows the ratio of Ni:Fe:P (15:7:78). The results suggest that the bonds between Ni and Fe were changed to metal-P bonds by phosphorization, forming heterogeneous metal-P mixtures (i.e., Ni_x_P_y_ and FeP). It is obviously reported that phosphide center of the metal phosphide becomes partially negative, making surface of metal positive^50^. Positive metal surface can more easily attract hydroxide ion and make UOR earlier, reducing its overpotential and increasing catalytic performance.

**Figure 2:**
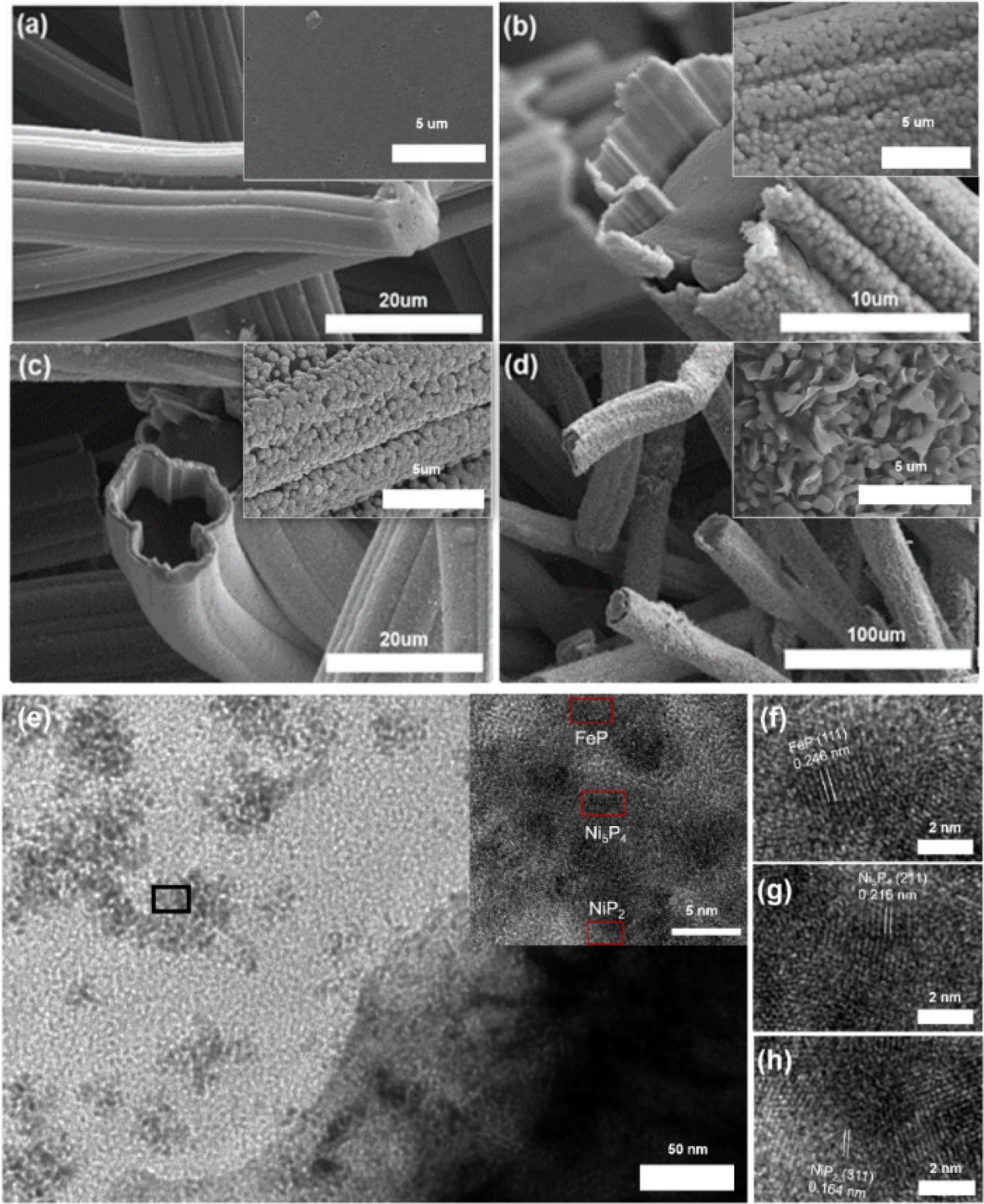
SEM micrographs of (**a**) CF, (**b**) Ni@CF, (**c**) Ni–Fe@CF, and (**d**) P-NiFe@CF with corresponding magnified images; and (**e**–**h**) HRTEM images of P-NiFe@C.

The SEM micrographs of the Ni@CF, Ni–Fe@CF, and P-NiFe@CF samples are shown in Fig. 2. The pristine CF comprised carbon fibers with a diameter of ~ 16 µm, forming an open network 3D structure. As seen in Fig. 2b,c, the individual fibers were completely coated with a thin layer of Ni_x_P_y_ and/or FeP as evidenced by EDX (Suppl. Figs. S1 and S2) and XPS analysis (Fig. 1). The high-resolution image (inset of Fig. 2a–d) revealed a rough surface with granular metal deposits that coalesced to form a continuous thin film. After phosphidation at 550 °C, 3D flower-like P-NiFe architecture on CF was formed as shown in the inset of Fig. 2d. The structure of P-NiFe coated on CF was further characterized by HRTEM, as shown in Fig. 2e–h. The P-NiFe structure comprised Ni_5_P_4_, NiP_2_, and FeP phases, which were identified by their lattice parameters as shown in Fig. 2f–h, in line with the XRD analysis. This result suggests intimate contact and strong interactions between the Ni_5_P_4_, NiP_2_, and FeP species in the hybrid structure. Additionally, uniform distribution of Ni, Fe, and P in the P-NiF nanoparticles was observed by TEM elemental EDX mapping (Suppl. Fig. S3).

### Electrochemical properties

**Figure 3:**
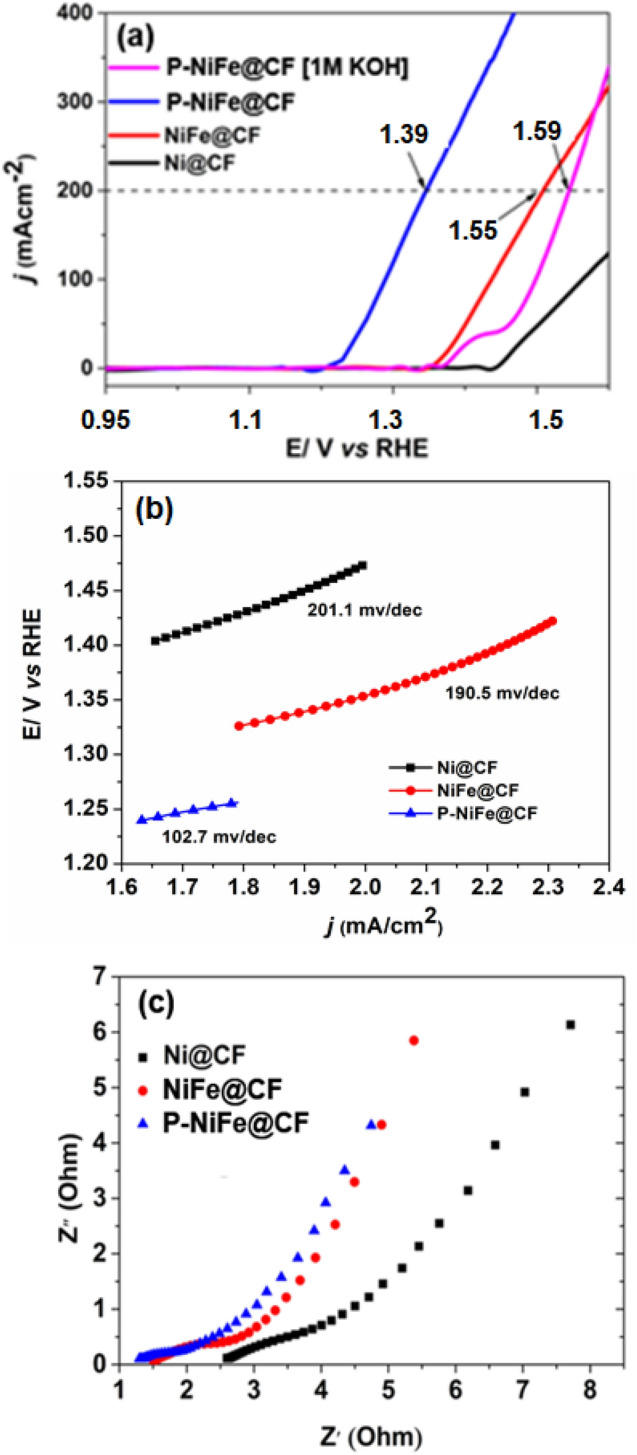
(**a**) LSV curves of Ni@CF, Ni–Fe@CF, and P-NiFe@CF in absence and presence of 0.33 M urea in 1.0 M KOH; (**b**) Tafel plots of different catalyst electrodes; and (**c**) Nyquist plots of different electrodes in 1.0 M KOH.

The electrochemical performances of Ni@CF, Ni–Fe@CF, and P-NiFe@CF catalyst electrodes in the UOR was analyzed by LSV using 1 M KOH and 0.33 M urea at a scan rate of 5 mV s^−1^, as depicted in Fig. 3. For comparison, bare carbon cloth (CC) and Pt on carbon cloth (Pt@CC) were also analyzed. The oxidation peak at 1.46 V in the LSV plot in 1 M KOH (Fig. 3a) was ascribed to the formation of active NiOOH sites for water oxidation^54,55^. As shown in Fig. 3a, the electrode potential of P-NiFe@CF for the UOR decreased considerably to 1.39 V to attain a current density of 200 mA cm^−2^ compared to 1.59 V for the OER (i.e., water oxidation), which indicates the oxidation current increased considerably in the presence of urea, thus indicating that H_2_ production by urea electrolysis was more energy efficient than water electrolysis. Figure 3a also shows the activities of the different catalysts for the UOR. Clearly, P-NiFe@CF required the lowest potential to attain a given current density for H_2_ production, indicating its superior UOR activity. Importantly, the electrochemical activity of P-NiFe@CF for the UOR exceeds that reported in the literature (Table S1). Additionally, the Tafel slope of P-NiFe@CF was 107.2 mV dec^−1^ which is much lower than those of Ni@CF and Ni–Fe@CF, as shown in Fig. 3b, further indicating faster kinetics of the UOR on P- NiFe@CF.

EIS measurements were conducted in a 1 M KOH solution to investigate the charge transfer rate (*Rct*) and double layer capacitance (*C*_*dl*_), as shown in Fig. 3c. The EIS spectra were fitted with an equivalent circuit, as shown in the inset of Suppl. Fig. S4. The P-NiFe@CF electrode exhibited a significantly reduced *R*_*ct*_ compared to Ni–Fe@CF and Ni@CF, indicating considerably enhanced charge transfer kinetics of the UOR on the P-NiFe@CF catalyst^56,57^. The smaller *R*_*ct*_ value of P-NiFe@CF was possibly due to the improved conductivity of metallic bonds such as Ni_5_P_4_, NiP_2_, and FeP, as the main factor. Furthermore, P-NiFe@CF featured the highest *C*_*dl*_ value, which was determined from the constant phase element value of the equivalent circuit (Suppl. Fig. S4), suggesting that P-NiFe@CF the highest electrochemically active surface area of the P-NiFe@CF^58^. This might be due to the higher valence state of Ni at the surface of P-NiFe@CF, as evidenced by the XPS analysis. Intimate contacts among the different crystal phases (Ni_5_P_4_, NiP_2_, and FeP) might also affect the electronic structure, making it more favorable for the UOR as previously reported based on empirical and computational approaches^59,60^. Additionally, the electrochemical stability of Ni–Fe–P@CF was higher than that of Ni@CF and Ni–Fe@CF (Suppl. Fig. S4), plausibly owing to the formation of the metallic phosphide-rich surface of P-NiFe@CF, which could resist structural collapse during the Ni^2+^ to Ni^3+^ transition^61^.

**Figure 4:**
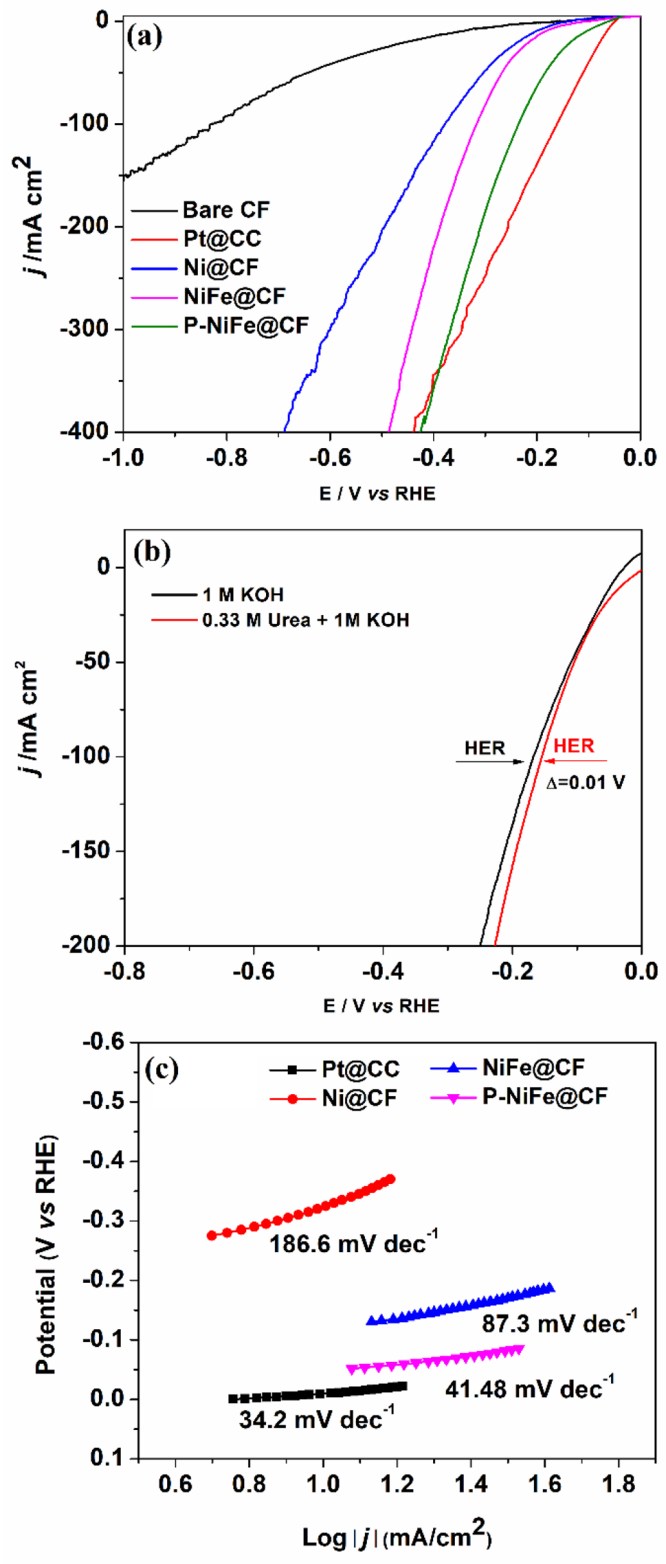
(**a**) LSV curves of different catalyst electrodes in 1.0 M KOH at a scan rate of 5 mV s^−1^, (**b**) LSV curves of P-NiFe@CF in 1.0 M KOH with and without 0.33 M urea, and (**c**) Tafel plots of different catalysts in 1.0 M KOH.

The HER performance of P-NiFe@CF was also analyzed by LSV in KOH with and without urea to examine its bifunctional catalytic activity for the UOR and HER. For comparative purposes, the HER activity of bare CF, Pt@CC, Ni@CF, and NiFe@CF was also studied for their HER performances, where the activity was indicated by the potential of the catalysts to achieve a given current density^2,4,62^. As evident from Fig. 4a, bare CF was inactive for the HER, with a negligible current density. The potential required for the Ni@CF electrode to attain 10 mA cm^−2^ was lowered from 0.124 to 0.104 V by Fe doping, and further considerably lowered to 65 mV by phospidation; however, it is still high than that (25 mV) of commercial Pt@CC. Additionally, P-NiFe@CF catalyst electrode had the highest stability among the evaluated catalysts (Suppl. Fig. S5). Interestingly, upon adding 0.33 M urea, a negative shift of only 10 mV was observed at a current density of 100 mA cm^−2^ (Fig. 4b), indicating that urea had little impact on the electrocatalytic activity for the HER. Figure 4c illustrates the Tafel plots of Pt@CC, P-NiFe@CF, NiFe@CF, and Ni@CF for the HER; demonstrating that P-NiFe@CF had a considerably lower Tafel slope of 41.4 mV dec^−1^ than NiFe@CF and Ni@CF, which is comparable with that (34.2 mV dec^−1^) of Pt@CC.

**Figure 5:**
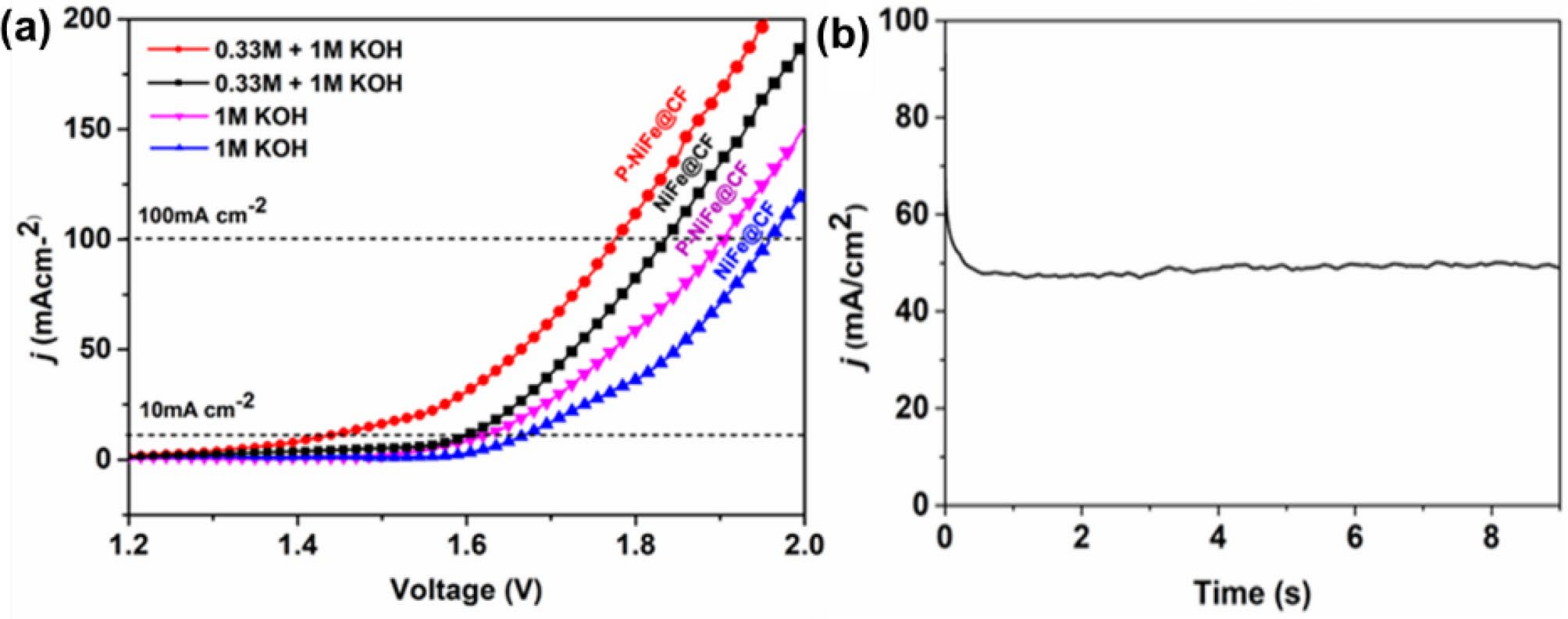
(**a**) Polarization curve of NiFe@CF and P-NiFe@CF in 1 M KOH with urea and without urea, and (**b**) a stability test in 1 M KOH and 0.33 M urea solution using P-NiFe@CF as anode and cathode at an applied potential corresponding to a current of 50 mA cm^−2^.

Two electrode urea electrolysis cells were constructed using the bifunctional catalyst electrodes as both the anode and cathode. As seen in Fig. 5a, the urea electrolyzer with P-NiFe@CF electrodes featured a current density of 10 mA cm^−2^ at a cell voltage of 1.37 V, which is lower than that (1.61 V) of the cell with Ni–Fe@CF. Furthermore, a current of 100 mA cm^−2^ in the urea electrolyzer with P-NiFe@CF electrodes was achieved at a low cell voltage of 1.72 V, which exceeds those reported for Ni_3_N/NF^63^, Ni_3_N NA/CC^64^, Ni_2_P/CC^65^, MoS_2_/Ni_3_S_2_^66^, Fe_11.1%_-Ni_3_S_2_/NF^31^, and Mo-NiP_2_^67^, as summarized in Suppl. Table S2. The P-NiFe@CF-based urea electrolyzer also exhibited good long-term electrochemical stability, as the current density remained stable for 8 h of operation at an applied voltage of 1.65 V after the initial drop due to concentration polarization^61^ (Fig. 5b).

## Conclusions

Bimetallic NiFe phosphides coated on CF by electroplating were successfully demonstrated to be efficient, free-standing, and bifunctional catalyst electrodes for the UOR and HER. P-NiFe@CF comprised Ni_5_P_4_, NiP_2_, and FeP crystal phases, forming metallic bonds and partially oxidized surface Ni. The bifunctional electrocatalytic activity of the Ni-based catalyst for the UOR and HER was improved by Fe doping, and was further considerably enhanced by phosphodation, where the activity is outstanding compared to the literature reports. The electrolysis cell with P-NiFe@CF as both the anode and cathode required only 1.42 V (*vs.* RHE) to attain a current density of 10 mA cm^−2^, with good electrochemical stability. The results indicate that urea electrolysis is an energy-efficient method for hydrogen production as compared to water splitting, and bimetallic NiFe phosphides coated on CF can be used as an efficient free-standing bifunctional catalyst for the UOR and HER in urea electrolysis.

## Supplementary Information

Supplementary Information.

## Data Availability

The datasets generated during and/or analysed during the current study are available from the corresponding author on reasonable request.
