# Supplementary material for: Ni–Fe phosphide deposited carbon felt as free-standing bifunctional catalyst electrode for urea electrolysis
Source: Sci Rep. 2021 Nov 9;11:22003. doi: 10.1038/s41598-021-01383-3 (PMC8578333; doi:10.1038/s41598-021-01383-3)
Supplement: Supplementary file 1 — Supplementary Information. [file 41598_2021_1383_MOESM1_ESM.docx]

*Supporting information for*

**Ni-Fe phosphide deposited carbon felt as free-standing bifunctional catalyst electrode for urea electrolysis**

Woo Hyun Yun, Gautam Das, Bohyeon Kim, Bang Ju park, Hyon Hee Yoon, and Young Soo Yoon

*^
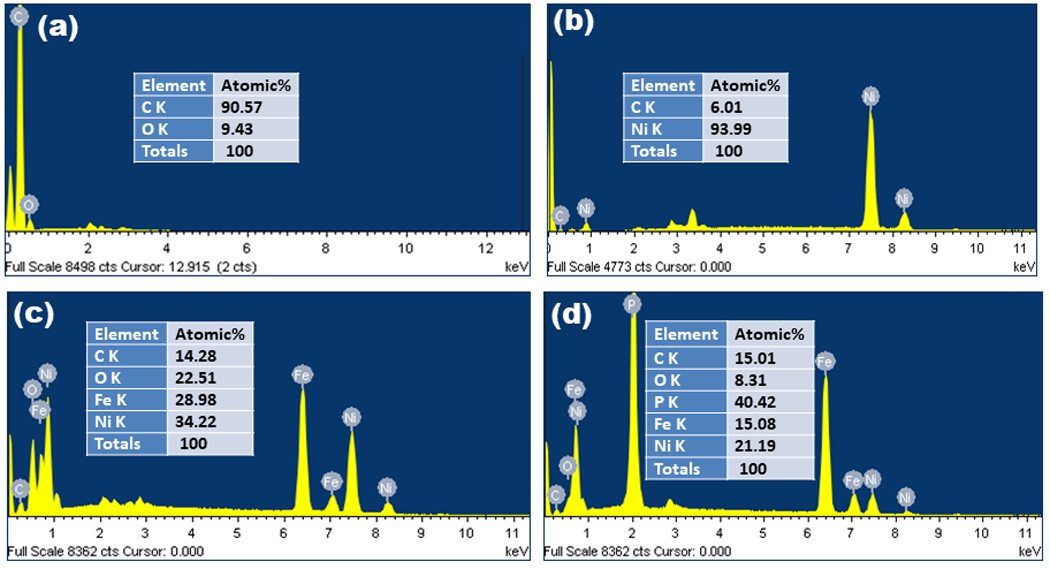
^*

**Fig. S1.** SEM EDX spectra of (a) carbon felt, (b) Ni@CF (c) NiFe@CF and (d) P-NiFe@CF.


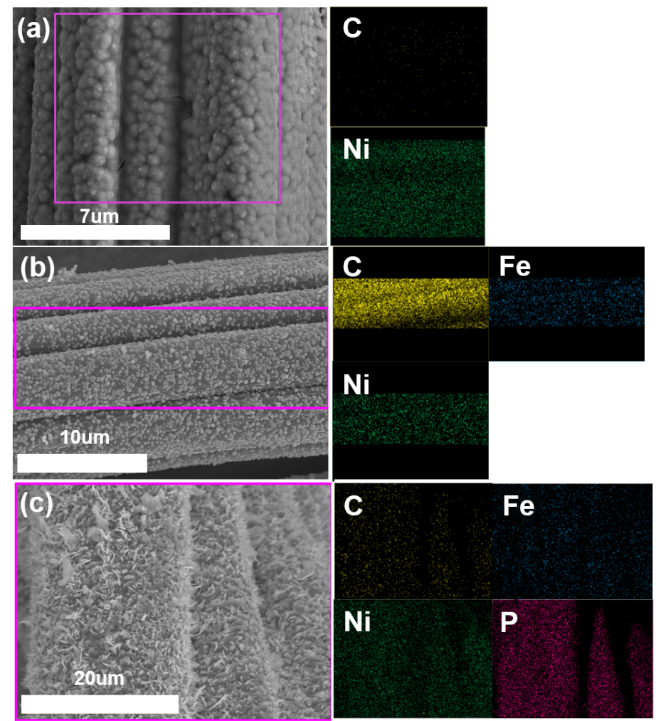


**Fig. S2.** EDS elemental mapping of (a) Ni@CF, (b) NiFe@CF (c) P-NiFe@CF obtained by SEM.


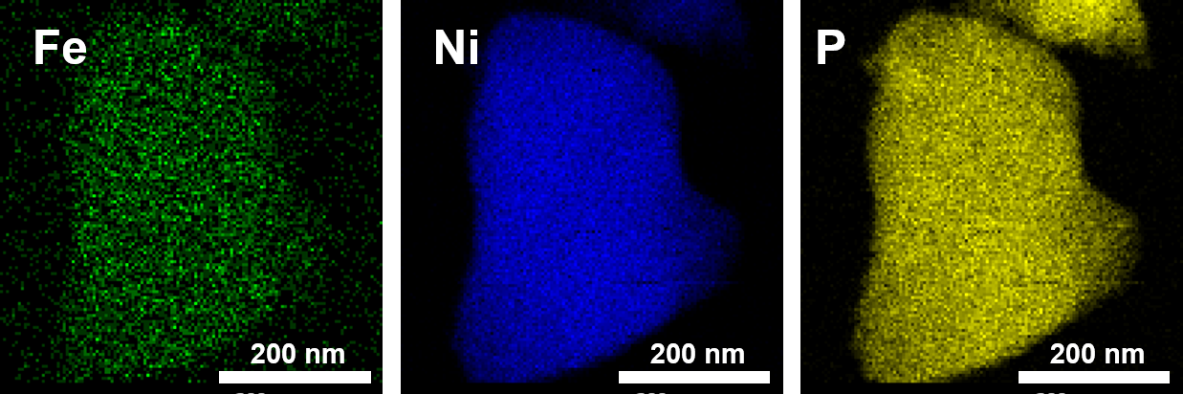


**Fig. S3.** EDS mapping of P-NiFe@CF obtained by HRTEM.


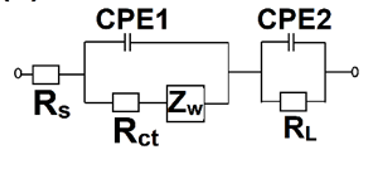

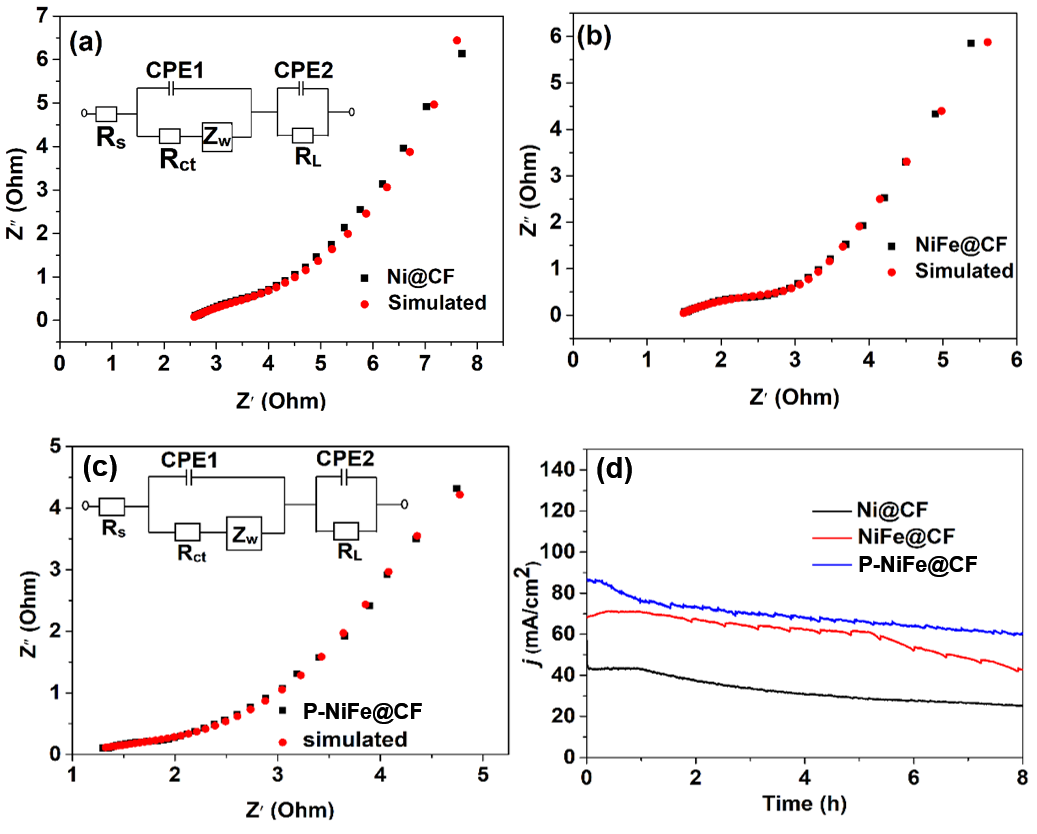


**Fig. S4.** Impedance plots of (a) Ni@CF, inset: equivalent circuit diagram, (b) NiFe@CF, and (c) P-NiFe@CF. (d) The stability test using 1 M KOH and 0.33 M urea as a electrolyte towards UOR.


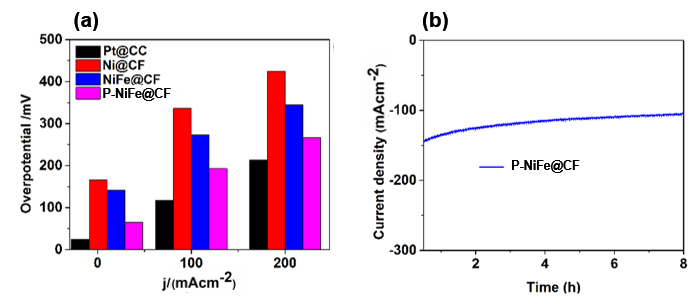


**Fig. S5.** (a) Bar graph illustrating overpotential *vs* current density plots of different catalysts determined from the LSV plot of HER in 0.33 M urea +1.0 M KOH and (b) the stability test using 1 M KOH and 0.33 M urea as an electrolyte towards HER.

**Table S1.** Performance comparison for urea electro-oxidation with different catalysts.

| Catalyst | Potential at 10 mAcm^-2^ (V *vs* RHE) | Electrolyte | Reference |
| --- | --- | --- | --- |
| NiO nanosheet array | 1.38 | 1 M KOH+ 0.33 M Urea | [1] |
| NiMoS | 1.38V | 1M KOH + 0.5M | [2] |
| Ni_3_N/NF | 1.34 | 1 M KOH+ 0.5 M Urea | [3] |
| Ni_3_N NA/CC | 1.35 | 1 M KOH+ 0.33 M Urea | [4] |
| Ni0-rich Ni/NiO | 1.33 | 1 M KOH+ 0.33 M Urea | [5] |
| Ni_2_P/CFC | 1.34 | 1 M KOH+ 0.33 M Urea | [6] |
| CoS_2_ NA/Ti | 1.40 | 1 M KOH+ 0.33 M Urea | [7] |
| MnO_2_/MnCo_2_O_4_/Ni | 1.33 | 1 M KOH+ 0.5 M Urea | [8] |
| Ni(OH)_2_ nanocube | 1.55 | 1 M KOH+ 0.33 M Urea | [9] |
| Graphene Ni(OH)_2_ | 1.52 | 5 M KOH+ 0.5 M Urea | [10] |
| a-Ni_2_P/G | 1.28 | 1 M KOH+ 0.5 M Urea | [11] |
| Ni_2_P/Fe_2_P/nickel foam | 1.36 V | 1 M KOH+ 0.5 M Urea | [12] |
| Ni_12_P_5_/Ni-Pi/NF | 1.37 | 1 M KOH+ 0.5 M Urea | [13] |
| NiFe@CF | 1.42 | 1 M KOH+ 0.33 M Urea | This work |
| P-NiFe@CF | 1.28 | 1 M KOH+ 0.33 M Urea | This work |

**Table S2.** Performances comparison for urea electrolysis in two electrode cell test with different catalysts.

| Anode catalyst | Cathode catalyst | Cell Potential  at 10 mA cm^-2^ | Electrolyte | Reference |
| --- | --- | --- | --- | --- |
| HC-NiMoS/Ti | HC-NiMoS/Ti | 1.59 | 1M KOH + 0.5M urea | [2] |
| Ni_3_N NA/CC | Ni_3_N NA/CC | 1.44 | 1M KOH + 0.33M urea | [4] |
| NF@Acid-H_2_ | NF@Acid-H_2_ | 1.49 | 1M KOH + 0.5M urea | [5] |
| CoS_2_ Na/Ti | CoS_2_ Na/Ti | 1.59 | 1M KOH + 0.3M urea | [7] |
| MnO_2_/MnCo_2_O_4_ | MnO_2_/MnCo_2_O_4_ | 1.55 | 1M KOH + 0.5M urea | [8] |
| Fe_11.1%_-Ni_3_S_2_/NF | Fe_11.1%_-Ni_3_S_2_/NF | 1.46 | 1M KOH + 0.33M urea | [14] |
| MoS_2_/Ni_3_S_2_ | MoS_2_/Ni_3_S_2_ | 1.45 (@20) | 1M KOH + 0.33M urea | [15] |
| NiFe@CF | NiFe@CF | 1.56 | 1 M KOH+ 0.33 M Urea | This work |
| P-NiFe@CF | P-NiFe@CF | 1.424 | 1 M KOH+ 0.33 M Urea | This work |

**References**

[1] Wu MS, Lin GW, Yang RS. Hydrothermal growth of vertically-aligned ordered mesoporous nickel oxide nanosheets on three-dimensional nickel framework for electrocatalytic oxidation of urea in alkaline medium. J Power Sources 2014;272:711–8. https://doi.org/10.1016/j.jpowsour.2014.09.009.

[2] Wang X, Wang J, Sun X, Wei S, Cui L, Yang W, et al. Hierarchical coral-like NiMOS nanohybrids as highly efficient bifunctional electrocatalysts for overall urea electrolysis. Nano Res 2018;11:988–96. https://doi.org/10.1007/s12274-017-1711-3.

[3] Hu S, Feng C, Wang S, Liu J, Wu H, Zhang L, et al. Ni 3 N/NF as Bifunctional Catalysts for Both Hydrogen Generation and Urea Decomposition. ACS Appl Mater Interfaces 2019;11:13168–75. https://doi.org/10.1021/acsami.8b19052.

[4] Liu Q, Xie L, Qu F, Liu Z, Du G, Asiri AM, et al. A porous Ni3N nanosheet array as a high-performance non-noble-metal catalyst for urea-assisted electrochemical hydrogen production. Inorg Chem Front 2017;4:1120–4. https://doi.org/10.1039/c7qi00185a.

[5] Zhang B, Wang S, Ma Z, Qiu Y. Ni0-rich Ni/NiO nanocrystals for efficient water-to‑hydrogen conversion via urea electro-oxidation. Appl Surf Sci 2019;496:143710. https://doi.org/10.1016/j.apsusc.2019.143710.

[6] Zhang X, Liu Y, Xiong Q, Liu G, Zhao C, Wang G, et al. Vapour-phase hydrothermal synthesis of Ni2P nanocrystallines on carbon fiber cloth for high-efficiency H2 production and simultaneous urea decomposition. Electrochim Acta 2017;254:44–9. https://doi.org/10.1016/j.electacta.2017.09.097.

[7] Wei S, Wang X, Wang J, Sun X, Cui L, Yang W, et al. CoS2 nanoneedle array on Ti mesh: A stable and efficient bifunctional electrocatalyst for urea-assisted electrolytic hydrogen production. Electrochim Acta 2017;246:776–82. https://doi.org/10.1016/j.electacta.2017.06.068.

[8] Xiao C, Li S, Zhang X, MacFarlane DR. MnO2/MnCo2O4/Ni heterostructure with quadruple hierarchy: A bifunctional electrode architecture for overall urea oxidation. J Mater Chem A 2017;5:7825–32. https://doi.org/10.1039/c7ta00980a.

[9] Wu MS, Ji RY, Zheng YR. Nickel hydroxide electrode with a monolayer of nanocup arrays as an effective electrocatalyst for enhanced electrolysis of urea. Electrochim Acta 2014;144:194–9. https://doi.org/10.1016/j.electacta.2014.08.098.

[10] Miller AT, Hassler BL, Botte GG. Rhodium electrodeposition on nickel electrodes used for urea electrolysis. J Appl Electrochem 2012;42:925–34. https://doi.org/10.1007/s10800-012-0478-1.

[11] Yan L, Sun Y, Hu E, Ning J, Zhong Y. Zhang Z, Hu Y. Facile in-situ growth of Ni2P/Fe2P nanohybrids on Ni foam for highly efficient urea electrolysis. J Colloid Interf. Sci. 541;2019, 279-286. <https://doi.org/10.1016/j.jcis.2019.01.096>.

[12] Xu X, Du P, Guo T, Zhao B, Wang, H. Huang, M. Design and Synthesis of Highly Performing Bifunctional Ni-NiO-MoNi Hybrid Catalysts for Enhanced Urea Oxidation and Hydrogen Evolution Reactions. ACS Sustainable Chem. Eng. 2020; 8;19;7463–7471. <https://doi.org/10.1021/acssuschemeng.0c01637>

[13] Xu, X. Du, P, Guo T. Zhao B. Wang H. Huang M. In situ Grown Ni phosphate@Ni12P5 Nanorod Arrays as a Unique Core–Shell Architecture: Competitive Bifunctional Electrocatalysts for Urea Electrolysis at Large Current Densities. ACS Sustainable Chem. Eng. 2020; 8; 19; 7463–747. <https://doi.org/10.1021/acssuschemeng.0c01814>.

[14] Zhu W, Yue Z, Zhang W, Hu N, Luo Z, Ren M, et al. Wet-chemistry topotactic synthesis of bimetallic iron-nickel sulfide nanoarrays: An advanced and versatile catalyst for energy efficient overall water and urea electrolysis. J Mater Chem A 2018;6:4346–53. https://doi.org/10.1039/c7ta10584c.

[15] Li F, Chen J, Zhang D, Fu WF, Chen Y, Wen Z, et al. Heteroporous MoS2/Ni3S2 towards superior electrocatalytic overall urea splitting. Chem Commun 2018;54:5181–4. https://doi.org/10.1039/c8cc01404c.
